# The 15th Anniversary of *Life*—Sepsis Trials

**DOI:** 10.3390/life15101517

**Published:** 2025-09-26

**Authors:** Jean-Louis Vincent

**Affiliations:** Department of Intensive Care, Erasme Hospital, Hôpital Universitaire de Bruxelles (HUB), Universite Libre de Bruxelles (ULB), 1070 Brussels, Belgium; jlvincent@intensive.org

**Keywords:** heterogeneity, personalization, biomarkers, subphenotypes

## Abstract

Clinical trials of drugs specifically targeting the sepsis response have frequently produced negative or inconclusive results. This has largely been due to the broad heterogeneity of enrolled patient populations, particularly when inclusion was based on the presence of the non-specific systemic inflammatory response syndrome (SIRS) criteria. The heterogeneity may have diluted any possible treatment effect: while some patients may have benefited from the intervention under investigation, others will have been harmed, resulting in an overall null-effect. Furthermore, an underlying infection is not always required for immune-modulating interventions to be effective; for example, patients with severe acute pancreatitis, but no infection, may still benefit from such therapies. There is therefore a need for better patient stratification or subphenotyping to identify those most likely to benefit from a particular therapy. Several trials have already adopted this approach using prognostic and/or predictive enrichment strategies. For example, measurements of triggering receptor expressed on myeloid cells (TREM) could be used to identify candidates most likely to respond to anti-TREM therapies, or patients with coagulopathy or specific inflammatory patterns could be selected for treatments like thrombomodulin or activated protein C. However, challenges remain, including the need for more rapid tools that can be used at the bedside to inform real-time treatment decisions given the rapidly evolving nature of the sepsis response. Nevertheless, the era of broad-spectrum “sepsis drugs” is now giving way to more selective, personalized interventions tailored to individual biological profiles, offering a more promising pathway for future therapeutic development—even in the absence of infection.

## 1. Introduction

Sepsis remains a major cause of morbidity and mortality worldwide, accounting for over 11 million deaths annually [1]. Despite decades of research and numerous clinical trials, there are still no specific, universally effective interventions for sepsis, and management essentially relies on infectious source removal and organ support. There are many possible reasons for the lack of success in identifying effective interventions, including the focus on mortality as an endpoint, the often delayed start of the intervention in the course of sepsis, and the fact that many therapeutic targets are also essential components of the host’s conserved immune response. However, the major contributor has been the heterogeneity of the patients included in the trials. Sepsis is not a single disease entity, but a complex clinical syndrome of life-threatening organ dysfunction caused by a dysregulated host response to infection and involving multiple overlapping pathways, including inflammatory, immune, coagulation, and metabolic [2]. Moreover, sepsis can affect individuals of any age, with different genetic backgrounds, comorbid conditions, and treatments; can be caused by different pathogens at different anatomical sites; can present at various stages of illness; and can be associated with different types and degrees of organ dysfunction. As such, targeting a single aspect of the biological response will never be effective in clinical trials that include broad populations of patients with the umbrella diagnosis of sepsis.

This review explores the trajectory of sepsis trials over time and the move from an approach based on single strategies in large heterogeneous groups to more targeted, personalized medicine using subphenotyping.

## 2. Early Clinical Trials in Sepsis

In the early phase of sepsis investigation, identification of endotoxin as a trigger of the sepsis response led to several clinical studies investigating the role of endotoxin blockade or neutralization, for example, using the human monoclonal antibody against endotoxin, HA-1A [3]. However, not all patients with sepsis have raised endotoxin levels; moreover, endotoxin is not the only player in the sepsis response. As a result, trials using this anti-endotoxin approach in large heterogeneous groups of patients with sepsis were largely negative. As research began to identify more sepsis pathways and components of the immune response, many became prospective therapeutic targets in efforts to develop an effective intervention against this often fatal disease. There followed a multitude of clinical trials targeting single inflammatory cytokines, such as tumor necrosis factor-alpha (TNF-α) [4] and interleukin-1 (IL-1) [5], as well as downstream pathways like nitric oxide (NO) production [6] and coagulation activation [7]. Unfortunately, almost all of these trials failed to consistently demonstrate a survival benefit in large studies and, in some cases, revealed harm.

## 3. Reasons for Failure: The Key Role of Heterogeneity

As noted earlier, patients with sepsis are highly heterogeneous with substantial differences in underlying characteristics, including age and genetic makeup; comorbid conditions; type of infection; state and degree of immune response; and treatment preferences. This diverse group of patients have often been bracketed together for clinical trials under the general label ‘sepsis’. Indeed, for many years, the definition of sepsis relied almost exclusively on the systemic inflammatory response syndrome (SIRS) criteria [8]—non-specific parameters such as elevated heart rate, fever, tachypnea, and leukocytosis that can occur in many critically ill patients, with or without sepsis. Using SIRS as a basis for patient inclusion in trials of sepsis interventions thus led to highly heterogeneous study populations. Later attempts to refine definitions did little to reduce this variability, largely because they failed to capture underlying biological or immunological states [2,9]. Many clinical trials in sepsis therefore continue to enroll diverse groups of patients based on non-causal syndromic definitions. As a result, any true therapeutic benefit in a subset of patients may be diluted or obscured by neutral or even harmful effects in others. Subgroup analyses in these trials have also frequently failed to demonstrate efficacy, in large part because stratification has typically relied on non-biological factors such as infection source, illness severity scores, or demographic variables, which may not reflect underlying pathophysiological differences or treatment responsiveness.

With improved understanding of the host response in sepsis, the multiple patient- and response-related components that contribute to the syndrome have become increasingly apparent. Patients differ widely in their immune response profiles, ranging from hyperinflammation to immunosuppression, and these responses evolve and change over time. Importantly, the efficacy and potential harm of therapeutic interventions can be influenced by these factors. It is therefore no longer appropriate to treat all patients with “sepsis” as a single, uniform group; rather, subphenotypes of sepsis need to be identified so that treatments can be tailored to each patient’s specific pathophysiological state, much as is already being done successfully in the field of oncology [10].

## 4. Subphenotyping in Sepsis

The terminology around sepsis stratification—including terms like subgroup, phenotype, subphenotype—can be confusing. For the purposes of this review, we will use the definitions provided by a recent consensus report [11]. A phenotype thus refers to a set of clinical characteristics or features that creates a recognizable pattern, e.g., sepsis, acute respiratory distress syndrome. A subgroup refers to a set of patients within a phenotype who can be grouped according to a pre-defined cut-off point in a clinical variable, e.g., the mild, moderate, and severe subgroups of ARDS based on the PaO_2_/FiO_2_ ratio [12]. A subphenotype refers to a biologically or clinically distinct subpopulation within a broader phenotype and is typically identified using more detailed analytical data—such as cytokine levels, gene expression profiles, or metabolomic patterns.

### 4.1. Clinical Approaches

Most attempts to stratify patients with sepsis have been based on clinical characteristics and variables. For example, Seymour et al. [13] used computer algorithm clustering and latent class analysis of 29 routinely available clinical variables, including demographic variables, vital signs, markers of inflammation (e.g., white blood cell count, C-reactive protein), markers of organ dysfunction or injury, and serum levels of glucose, sodium, hemoglobin, chloride, bicarbonate, lactate, and albumin. Four distinct clinical subphenotypes of sepsis were identified—α, β, γ, and δ—which differed significantly in terms of demographics, laboratory abnormalities, organ dysfunction patterns, and outcomes. Importantly, these subphenotypes could not be described by traditional patient subgroups based on site of infection or severity of illness scores. Moreover, when used in simulations of several ‘negative’ clinical trials (of early goal-directed therapy, activated protein C, and eritoran) the subphenotypes showed differential treatment effects, supporting a role of more precise patient classification in sepsis trials. Other groups have performed similar analyses on routine clinical and biological variables and identified 2–5 different sepsis subphenotypes, which have been associated with different outcomes [14,15,16,17,18] and some also with different treatment responses, e.g., to corticosteroid [15] or fluid [17] therapy. However, clinical-based subphenotypes, although comparatively easy to obtain and indicative of a patient’s physiological status, do not capture the underlying biological or molecular mechanisms driving sepsis.

### 4.2. Omics-Based Approaches

Over the past few decades, advances in molecular biology techniques, including next-generation sequencing and mass spectrometry, completion of the human genome project, increased computing power and accessibility, and growing availability of large amounts of recorded patient data, have enabled large-scale, data-driven analysis of genes, proteins, and metabolites to help improve understanding of complex systems such as sepsis. These omics approaches have enabled subphenotypes of sepsis to be identified based on gene expression profiles, plasma cytokine levels, and metabolic signatures. As for the clinical-based subphenotypes discussed earlier, these subphenotypes have been associated with different outcomes and treatment responses. For example, Davenport et al. identified two distinct sepsis response signatures (SRS1 and SRS2) using a transcriptomic analysis of peripheral blood leukocytes. SRS1 was present in patients with an immunosuppressed subphenotype, and associated with higher mortality than the SRS2 subphenotype [19]. Using these classifications in a post hoc analysis of a randomized controlled trial of steroid therapy in septic shock revealed treatment-effect heterogeneity, with hydrocortisone use associated with increased mortality in those with the immunocompetent SRS2 subphenotype [20]. Also using transcriptomic analysis, Scicluna et al. [21] identified four subphenotypes, designated Mars1–4. Patients with Mars1 had the highest mortality rates, and this was associated with a decrease in innate and adaptive immune genes. The Mars3 subphenotype, associated with a lower mortality than Mars1, had increased expression of adaptive immune or T-cell functions. Using unsupervised transcriptomic analysis, Sweeney et al. identified three clusters, which they interpreted as sepsis phenotypes; these were named inflammopathic, adaptive, and coagulopathic, based on the predominant biological mechanism present, i.e., activation of the innate immune system, activation of the adaptive immune system, and activation of the coagulation system, respectively [22]. Interestingly, these subphenotypes had significant overlaps with some of the subphenotypes identified in the study by Davenport and colleagues [19]. Using a metabolomics approach, Rogers et al. [23] identified three subsets of sepsis patients with distinct metabolic profiles, driven largely by differences in lipid metabolites. Patients in the group with the lowest lipid levels had the highest mortality rates. Similarly using metabolomics, Antcliffe et al. [24] identified three subphenotypes of patients with septic shock, again largely defined by differences in lipid species. Of note, the subphenotype to which a patient belonged changed over time, reflecting the dynamic nature of sepsis; in that study, persistence of a subphenotype associated with high lipid levels was associated with survival [24].

Although omics approaches can thus reveal biologically distinct subphenotypes in sepsis by identifying complex molecular signatures, these techniques are expensive and time-consuming, and are not yet widely used in clinical practice.

## 5. Using Subphenotyping for Trial Enrichment to Reduce Population Heterogeneity

The “prospective use of any patient characteristic to select a study population in which detection of a drug effect (if one is in fact present) is more likely than it would be in an unselected population” is known as enrichment [25]. Essentially, trial enrichment involves the careful selection of participants—based on one or more clinical, demographic, or biological markers—to reduce heterogeneity among study populations and increase the likelihood of observing a true treatment effect. There are three main types of enrichment: general, prognostic, and predictive. General enrichment involves simple strategies aimed at decreasing variability, such as excluding patients who are more likely to experience adverse effects from the intervention, for example, older adults, individuals with known contraindications to the study drug or its components, or those with a limited life expectancy. This type of enrichment is already widely used and applied to some extent in most trials. We will discuss the other two types: prognostic and predictive enrichment.

### 5.1. Prognostic Enrichment

Prognostic enrichment involves selecting patients who have a greater baseline risk of developing a relevant endpoint (e.g., mortality) compared to other patients with the same condition [25,26]. Prognostic enrichment can increase the absolute treatment effect, but will not alter the relative treatment effect. An example of prognostic enrichment is the MONARCS trial [27], which evaluated afelimomab, an anti-TNF-α antibody, in patients with sepsis. In this trial, patients were stratified according to levels of interleukin-6 (IL-6), a marker associated with worse outcomes in sepsis. The results showed a modest but statistically significant reduction in 28-day mortality and improved organ dysfunction in patients with elevated IL-6 levels.

### 5.2. Predictive Enrichment

With predictive enrichment, patients with a higher likelihood of responding to a specific intervention are selected, guided by an established biological mechanism [25,26]. In contrast to prognostic enrichment, predictive enrichment can increase the relative treatment effect by identifying a subset of patients in which the intervention is expected to be more effective. Predictive enrichment is closely linked to the concept of “treatable traits”—measurable, clinically relevant characteristics (such as biomarkers, molecular signatures, or disease pathways) that can be altered by one or more therapeutic interventions. Treatable traits thus not only help to define a patient subset, but also provide a direct rationale for the treatment choice [11]. Importantly, “treatable traits” are independent of syndromic definitions [11,28,29]. Different disease entities, e.g., severe acute pancreatitis, severe burn injury, severe trauma, although having distinct underlying causes, can give rise to similar immune responses and molecular patterns and may respond to similar immunomodulatory therapies [30,31,32].

Several studies targeting specific interventions for sepsis have already used predictive enrichment. For example, levels of soluble triggering receptor expressed on myeloid cells-1 (sTREM-1), which are elevated in patients with sepsis and have been associated with poor outcomes [33], were used in a phase 2 trial of nangibotide, a TREM-1 inhibitor, in patients with septic shock [34]. Although the primary efficacy endpoint was not met, there were clinically relevant beneficial effects on organ function in septic shock patients with the highest baseline sTREM-1 levels. As the intervention directly targets the TREM-1 pathway, this was an example of predictive enrichment targeting a treatable trait. Another example of predictive enrichment is the randomized trial of thrombomodulin versus placebo, in which patients were selected for inclusion based on the presence of sepsis-associated coagulopathy [35]. Finally, recent (unpublished) findings from the Tigris trial, which studied use of polymyxin B hemoadsorption in patients with septic shock and high endotoxin levels and showed a reduction in mortality in the treatment group, confirm the potential value of careful patient selection based on targeting a biological mechanism [36].

## 6. Challenges in Implementing Trial Enrichment

Although the ability to select patients for clinical trials who are more likely to have a disease-related event and/or respond to a particular intervention has the potential to reduce heterogeneity, increase the likelihood of detecting a treatment effect, and assist in the development of effective sepsis therapies, challenges remain regarding how this approach can be effectively and efficiently integrated into current trial design [11,26,29,37,38].

**Figure 1:**
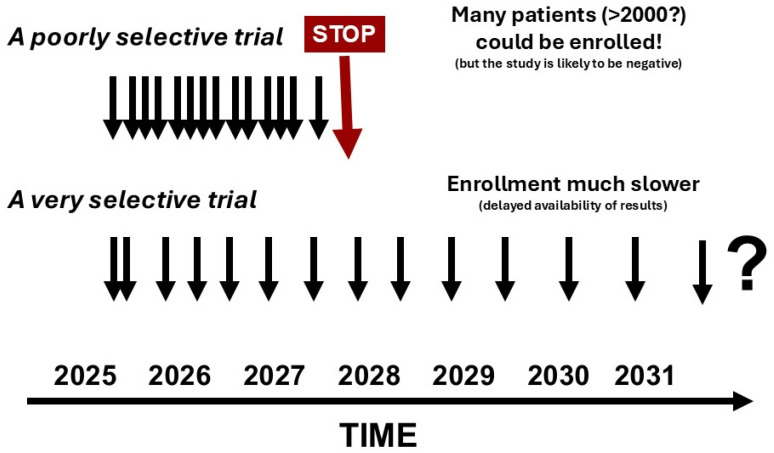
Simple schematic showing effects on patient enrollment with very selective recruitment. Each arrow symbolizes the inclusion of (a given number of) patients in the trial.

First, subphenotypes must be simple to identify, using universally available markers, and must be clinically validated across multiple diverse populations to ensure they are applicable in all settings [11]. Subphenotypes may be different in children and adults. Most studies in this field have been retrospective, used data from one or two cohorts with similar demographics, and been conducted primarily in high-resource settings. Increased international collaboration is needed to address this and facilitate prospective studies across continents to assess applicability also in low-resource settings [11]. Second, given the rapidly changing course of sepsis and the need to start or adjust treatments promptly, subphenotypes need to be identified rapidly—ideally in real-time—if they are going to be used for treatment decisions. Currently many such analyses take several hours if not days. Point-of-care platforms and rapid molecular diagnostics are in development but are not yet widespread in clinical use [39]. The more complex omics-based approaches to subphenotyping are expensive and technically demanding, creating additional challenges for repeated, rapid testing. Moreover, as this field evolves, studies will need to be conducted evaluating the cost-effectiveness of this approach. Third, patients may have several different subphenotypes or treatable traits simultaneously, making it difficult to determine which intervention(s) is optimal. Moreover, given the rapidly evolving biological nature of sepsis, subphenotypes can change during the disease course so that treatments may need to be adjusted. Van Amstel et al. [40] recently observed a transition from a hyperinflammatory to a hypoinflammatory subphenotype in about 50% of patients over a 4-day period. Such patients may benefit from immunosuppressive therapies early in the disease course, but immune-stimulating treatments may become more appropriate later, highlighting again the need for repeated monitoring of subphenotypes. Fourth, most studies have relied on blood-based sampling to identify potential biomarkers, but samples from other compartments may be more relevant in some patients, for example, from bronchoalveolar lavage fluid in acute respiratory distress syndrome (ARDS) [41], urine in acute kidney injury [42], or cerebrospinal fluid in acute brain injury. Expanding biomarker discovery and validation to include these compartments may improve the precision of enrichment strategies. Fifth, while enrichment strategies can help select patients more likely to respond to the therapy under investigation, if inclusion criteria are too selective, it can take longer to find and enroll enough patients (Figure 1). This can lead to delays in obtaining results, affect data interpretation as other treatment factors may have altered during the course of the study, dampen researcher enthusiasm as publications will be slow to achieve, and further slow recruitment because the smaller proportion of eligible patients reduces the focus on patient identification and inclusion. Additionally, industry interest may be limited, as companies want rapid returns on their investments.

Finally, using subphenotyping for clinical trial enrichment in sepsis may raise regulatory considerations regarding the generalizability of the trial results and whether efficacy demonstrated in a selected subgroup can support broader drug approval [25]. Regulatory agencies may also require validation that the subphenotype is clinically meaningful, reproducible, and identified using standardized methods.

## 7. Changing Clinical Trial Design

This shift toward trial enrichment necessitates a move from traditional fixed-group randomized controlled trials, and a move toward alternative trial designs—such as adaptive, basket, or platform trials—that can tailor interventions, randomize within defined subsets of patients, and adjust in real time based on emerging data. Several such trials have already been started, building on the experience and success of trials such as REMAP-CAP and RECOVERY during the COVID-19 pandemic [43]. The PALETTE trial (NCT06381661) is a treatable traits-guided, adaptive, Bayesian basket trial. Patients with sepsis are included based on the presence of a specific immunomodulation, coagulation, or corticosteroid treatable trait identified by different characteristics and biomarkers, and randomized to one of the relevant parallel experimental arms or a control group. For example, patients with the immunomodulation trait will be randomized to treatment with either tocilizumab, baricitinib, or anakinra. The TRAITS platform (ISRCTN82395639) expands the idea that treatable traits are syndrome-independent by including all patients with organ dysfunction requiring organ support, whether or not they have sepsis. They are then randomized to one of the treatment arms based on an identified treatable trait, currently either LYMP-RESP (clinical evidence of lymphopenia and respiratory dysfunction) and ENDO-SHOCK (clinical evidence of shock and inflammation). This platform has embedded biological sampling, which will enable the mechanisms of intervention effect to be studied and facilitate the discovery of new biomarkers and treatable traits.

Alongside changes in trial design, endpoints should also be reconsidered [11]. Traditional endpoints like 28-day mortality are often too non-specific to detect treatment effects within biologically distinct subsets of patients, and fail to reflect differences in disease trajectory, timing of risk, or mechanism of action [44,45]. Intermediate outcomes and subphenotype-specific endpoints—such as organ dysfunction resolution, immune recovery, or biomarker changes—should also be considered.

## 8. Conclusions

In a recent meta-analysis, almost 400 randomized controlled trials were identified in which immunomodulatory interventions had been tested in sepsis; only 8% had used a personalized approach to patient selection, using clinical or biological markers [46]. As subphenotyping—determination of a set of biological or clinical characteristics using detailed analytical data to identify distinct patient subgroups with shared pathophysiological mechanisms or treatment responses—becomes more widespread, the number of studies using a more personalized approach to target therapies will expand. This will increase the likelihood of identifying effective therapies for specific patient subgroups. It may also help optimize antibiotic therapy by distinguishing patients who truly need antibiotics from those who do not. Machine learning and other artificial intelligence tools will play key roles in integrating multidimensional (e.g., clinical, immune, microbiological, omics) data into meaningful patterns, enabling the identification of relevant subphenotypes and treatable traits (measurable, clinically relevant characteristics that can be altered by one or more therapeutic interventions). Until omics data can be successfully incorporated into the subphenotyping process—overcoming current limitations related to cost, processing time, and standardization—more accessible biomarker-guided trial enrichment remains a pragmatic approach to advancing sepsis management.

**Figure 2:**
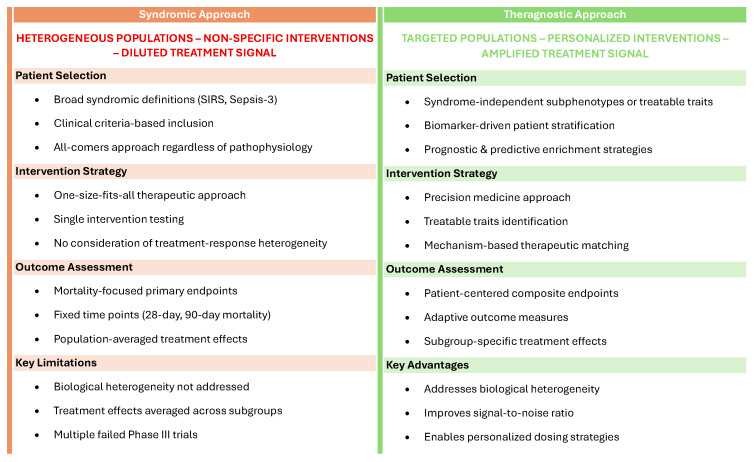
Comparison of general syndromic and theragnostic strategies in sepsis clinical trials.

Sepsis trials are likely to shift away from using traditional syndromic definitions towards a theragnostic approach, targeting interventions to specific patient groups identified by syndrome-independent subphenotypes or treatable traits, and using prognostic and/or predictive enrichment to guide patient inclusion (Figure 2).

Determining which subphenotypes are clinically relevant and building an evidence-base for subphenotype-guided treatments will require international collaboration across industry, researchers, and clinicians. More rapid subphenotyping techniques are needed to enable identification of changes in subphenotypes during the course of sepsis and allow therapies to be adjusted accordingly. The eventual goal is to provide a dynamic, personalized medicine approach for critically ill patients, which will result in improved outcomes.

## Data Availability

No new data were created or analyzed in this study. Data sharing is not applicable to this article.
